# Polymerization of Poly(3,4-ethylenedioxythiophene) on Sulfated Cellulose Nanofiber and Its Conducting Property

**DOI:** 10.3390/ma18061273

**Published:** 2025-03-13

**Authors:** Naofumi Takahashi, Atsuya Ogo, Takeshi Shimomura

**Affiliations:** Graduate School of Engineering, Tokyo University of Agriculture and Technology, 2-24-16 Naka-cho, Koganei 184-8588, Tokyo, Japan

**Keywords:** conducting polymer, bio-based polymer, PEDOT, cellulose nanofiber, sulfated cellulose

## Abstract

Recent research on incorporating biomass resources into functional polymers has garnered significant attention. Poly(3,4-ethylenedioxythiophene)/poly(styrene sulfonate) (PEDOT:PSS) is the most commercially successful conducting polymer composed of over 70 wt% petroleum-derived PSS, which presents an opportunity for partial replacement with biomass-based resources. In this study, a complex of PEDOT and sulfated cellulose nanofiber (PEDOT:s-CNF) was synthesized, and the relationship between its conductivity and doping conditions was investigated. PEDOT was synthesized on s-CNF, which was used in place of PSS, and the results indicate that conductivity increases as PEDOT polymerization progresses; however, excessive polymerization reduces electrical conductivity. Based on X-ray photoelectron spectroscopy and zeta potential measurements, the doping concentration decreases as PEDOT polymerization progresses to an excess state. This decrease is attributed to the depletion of sulfate groups, which act as dopants on s-CNFs, occurring as a consequence of the addition of PEDOT monomers. Enhancing the degree of sulfate group substitution on s-CNFs and incorporating additional dopants containing sulfonic groups improved conductivity. Specifically, adding *p*-toluenesulfonic acid (PTSA) as a dopant increased conductivity, reaching approximately 10 mS cm^−1^. However, at higher PTSA concentrations, the strong acidity of sulfonic groups reduced the degree of sulfate group dissociation, leading to a decline in doping efficiency.

## 1. Introduction

Conducting polymers have attracted considerable attention owing to their relatively low cost, flexibility, and high processability, including their suitability for coating and inkjet printing. Among them, poly(3,4-ethylenedioxythiophene)/poly(styrene sulfonate) (PEDOT:PSS) is the most commercially successful conducting polymer. Unlike other conducting polymers, PEDOT:PSS is stable when dispersed in water because it forms an ionic complex between hydrophobic PEDOT, which provides electrical conductivity, and hydrophilic PSS, which ensures stable dispersion in aqueous media. Although PSS is non-conductive, it plays a crucial role in imparting hydrophilic properties to PEDOT:PSS, enhancing its processability. Additionally, PSS contains sulfonic acid groups that serve as negatively charged dissociating groups, stabilizing the positive charge on the semiconducting PEDOT backbone and acting as an acceptor dopant. PEDOT:PSS has been reported to exhibit higher conductivity than other conducting polymers, exceeding 200 S cm⁻^1^ under secondary doping conditions [1].

To improve conductivity, various attempts have been made to replace PSS with alternative anions [2,3,4,5]. Crispin et al. replaced polymeric PSS with small anions, such as *p*-toluenesulfonic acid (PTSA), which contains a sulfonic acid group, to reduce the insulating volume introduced by polyanions [2]. PEDOT:PTSA films have demonstrated high electrical conductivity, exceeding 1000 S cm^−1^ [3]. Tumová et al. synthesized PEDOT doped with dodecylbenzenesulfonic acid (PEDOT:DBSA) for cell culture applications [4]. They reported that the conductivity of the thin films significantly improved when DBSA was used in the post-treatment phase. Lu et al. synthesized PEDOT complexes with anionic polysaccharides containing hydroxyl or carboxyl groups through electrochemical polymerization [5].

Recent studies have explored the effective utilization of biomass resources as an alternative to petroleum-derived materials. As the most abundant biopolymer, cellulose has gained attention as a potential substitute for PSS. In PEDOT:PSS, over 70 wt% of petroleum-derived PSS has the opportunity for partial substitution with cellulose. Replacing only the substitutable portion with biomaterials aligns with the goal of achieving carbon neutrality. Although there have been a considerable amount of reports on the PEDOT:PSS composed of cellulose and its excellent properties, including high conductivity [6,7,8,9,10], the process of mixing commercially available PEDOT:PSS is not sufficient for the suppression of petroleum substances, so the replacement of PSS with cellulose is a method that approaches carbon neutrality. Some studies have investigated the replacement of PSS with sulfated cellulose nano-/microcrystals [11,12,13]. Horikawa et al. synthesized PEDOT–cellulose using highly sulfated, partially crystalline cellulose through the chemical oxidative polymerization of 3,4-ethylenedioxythiophene (EDOT) in an aqueous solution [11]. The electrical conductivity of PEDOT–cellulose was reported to be 0.58 S cm^−1^ at a degree of sulfate group substitution (DS) of 1.03. Feng et al. synthesized PEDOT/sulfated nanocrystalline cellulose (SNC) and proposed its use in humidity sensors [12]. Owing to the negative charge from the sulfate groups on the cellulose chain and the colloidal stability of nanocellulose, PEDOT–cellulose films exhibited relatively high conductivity (~1 S cm^−1^). These high dispersions facilitated film deposition onto various substrates, and PEDOT:SNC printed on substrates demonstrated conductivity variations in response to external stimuli such as humidity and bending. Li et al. prepared PEDOT/microcrystalline cellulose through oxidative polymerization using different sulfated cellulose contents and evaluated its electrochemical performance [13].

In contrast to these studies using nano-/micro-cellulose, limited research has been conducted on PEDOT in combination with cellulose nanofibers (CNFs). CNF sulfation is typically achieved by mixing cellulose with sulfating reagents such as sulfuric acid [14], sodium sulfite [15], chlorosulfuric acid [16,17], or sulfur trioxide–pyridine complexes [18]. The sulfate groups on sulfated CNFs (s-CNFs) can be used as substitutes for PSS, while the long, persistent structure of CNFs may impart properties to conductive PEDOT that differ from those of the coiled conformation of PSS. Yoshida et al. synthesized various PEDOT/s–CNF thin films by adjusting the ratio of the EDOT monomer to CNFs [17]. Their PEDOT/s–CNF thin film exhibited a conductivity of 10.1 S cm^−1^, which was higher than that of PEDOT–cellulose materials, and demonstrated near-infrared (NIR) shielding effects.

Although PEDOT/sulfated cellulose (nanocrystals and nanofibers) is expected to exhibit higher conductivity than simple PEDOT:PSS–cellulose composites owing to the absence of non-conductive PSS, its conductivity remains significantly lower than that of pure PEDOT:PSS subjected to secondary doping. This reduction in conductivity is attributed to the doping state of the sulfated cellulose. Therefore, in this study, we synthesized PEDOT:s-CNF and examined the relationship between its doping state and conductivity, including the effect of additional doping with other sulfonic acids. In PEDOT:PSS, PSS molecules surround EDOT molecules during polymerization, serving dual roles as a water dispersant and a dopant for PEDOT; however, the specific role of s-CNF in this context remains unclear. A comprehensive understanding of the doping mechanism in PEDOT:s-CNF is necessary to enhance its conductivity.

## 2. Materials and Methods

### 2.1. Materials

Aqueous dispersions of sulfated cellulose nanofibers (s-CNF) (Stellafine^®^, 1.0 wt%) with different sulfur contents of 1.54 mmol g⁻^1^ and 1.91 mmol g⁻^1^ were purchased from Marusumi Paper Co., Ltd., (Ehime, Japan). The degree of substitution (DS) was defined as the number of sulfate groups per glucose unit, where the sulfur contents of 1.54 mmol g⁻^1^ and 1.91 mmol g⁻^1^ corresponded to DS = 0.277 and DS = 0.343, respectively. 3,4-Ethylenedioxythiophene (EDOT), potassium persulfate (KPS), and *p*-toluenesulfonic acid monohydrate were purchased from Tokyo Chemical Industry Co., Ltd., (Tokyo, Japan). Iron (III) sulfate and hydrochloric acid (HCl) were obtained from Fujifilm Wako Chemicals Co., (Osaka, Japan).

### 2.2. Preparation of PEDOT:s-CNF Dispersions

**Figure 1:**
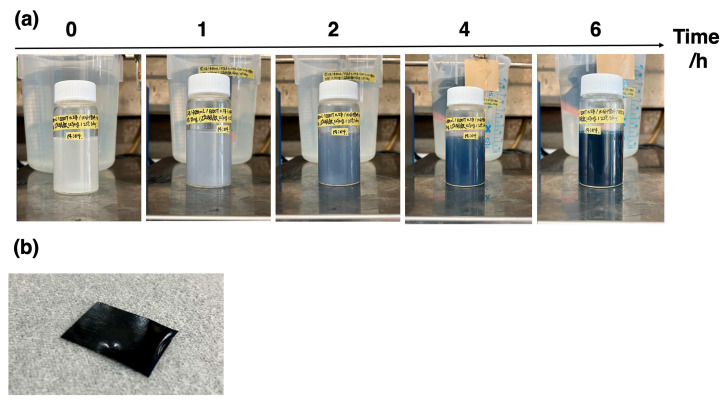
(**a**) Color change in the PEDOT:s-CNF dispersion during polymerization and (**b**) a photographic image of the PEDOT:s-CNF casting film.

Aqueous dispersions of s-CNF (20 g) and distilled water (60 g) were mixed, and the pH was adjusted to approximately 1 by adding HCl. To this pH-adjusted s-CNF dispersion, a controlled amount of EDOT, 72 mg of KPS as an initiator, and 500 μg of Iron (III) sulfate as an oxidizing agent were gradually added. The mixture was stirred continuously (200–300 rpm) for 24 h at 25 °C to allow for polymerization. PEDOT:s-CNF dispersions were synthesized by varying the mass ratio of EDOT to s-CNF, denoted as *m*(EDOT)/*m*(s-CNF), with values of 0.2, 0.6, 1, 2, 5, and 10. As polymerization progressed, the dispersion changed from turbid white to deep blue (Figure 1), indicating the polymerization of PEDOT. After polymerization, the solution was dialyzed in pure water for 72 h at room temperature while changing the water several times to remove unreacted impurities. The final concentration of the dispersion was adjusted to 0.6 wt%.

### 2.3. Characterization Methods

The polymerization of EDOT was confirmed using FT-IR (FT/IR-4100, JASCO Co., Tokyo, Japan) and UV-Vis spectrometer (V-630, JASCO Co). The morphology of the PEDOT:s-CNF films was examined via scanning electron microscopy (SEM) using a Merlin high-resolution field-emission SEM (S-4500, HITACHI Ltd., Tokyo, Japan).

X-ray photoelectron spectroscopy (XPS) was performed using a JPS-9030 spectrometer (JEOL Ltd., Tokyo, Japan), and spectral curve fitting was analyzed using SPECSURF Analysis software (v2.0, JEOL Ltd.).

Zeta potential measurements were conducted using a SZ-100Z Nanoparticle Analyzer (HORIBA, Ltd., Kyoto, Japan) by laser Doppler electrophoresis. The PEDOT:s-CNF concentration was maintained in the dilute region (0.06 wt%) during this measurement.

The electrical conductivity of PEDOT:s-CNF films was determined using a source measure unit (Keithley 236, Keithley Instruments, Inc., Cleveland, OH, USA) via the two-probe method in an argon-purged glove box. The mean conductivity was calculated using at least five independent measurements per sample.

## 3. Results and Discussion

### 3.1. Properties of PEDOT:s-CNF

**Figure 2:**
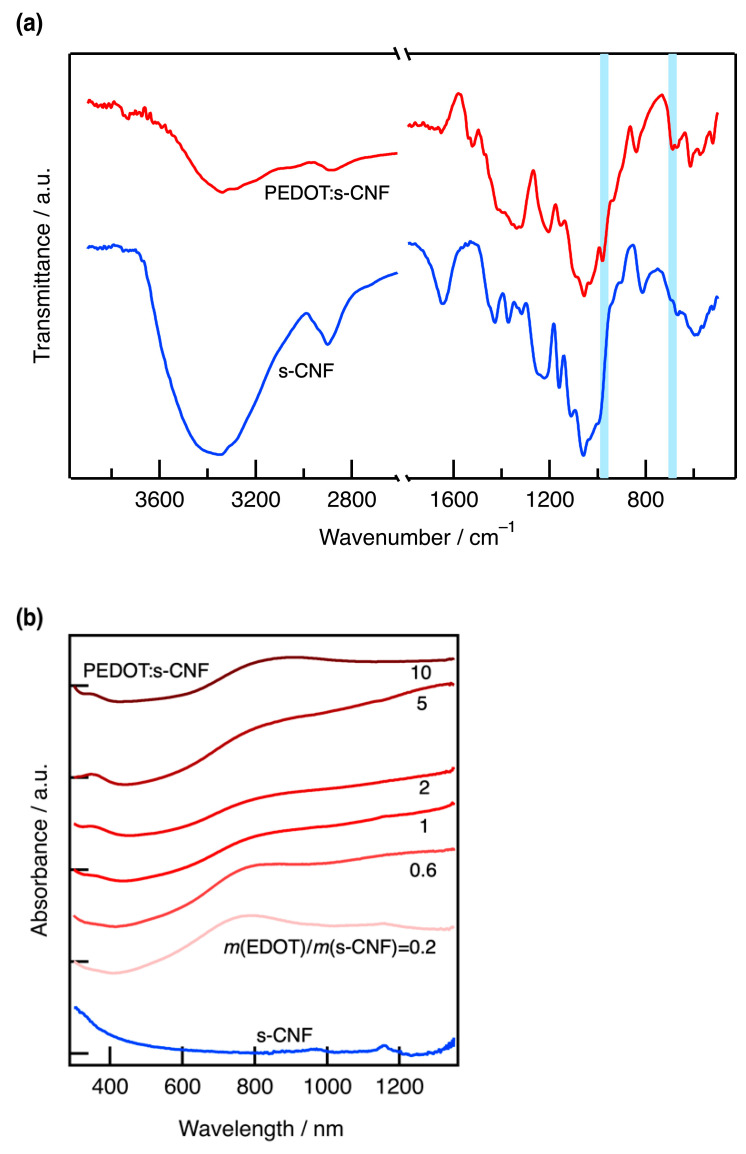
(**a**) FT-IR spectra of pristine s-CNF and PEDOT:s-CNF (DS of s-CNF = 0.277; *m*(EDOT)/*m*(s-CNF) = 0.6). (**b**) UV spectrum of PEDOT:s-CNF with varying *m*(EDOT)/*m*(s-CNF) from 0.2 to 10.0.

Figure 2a exhibits the FT-IR spectra of pristine s-CNF and PEDOT:s-CNF (DS of s-CNF = 0.277) and *m*(EDOT)/*m*(s-CNF) = 0.6. In addition to the characteristic absorption bands of s-CNF, PEDOT:s-CNF exhibited additional absorption bands at 973 cm⁻^1^ and 688 cm⁻^1^, which were attributed to the C-S-C bond in the thiophene ring [13]. Figure 2b displays the UV-Vis absorption spectra of aqueous dispersions of pristine s-CNF (DS = 0.277) and PEDOT:s-CNF with varying *m*(EDOT)/*m*(s-CNF) ratios from 0.2 to 10. All PEDOT:s-CNF spectra exhibited a broad absorption band at around 800 nm, which differed from that of s-CNF. The broad peak at approximately 800 nm, along with an absorption tail extending into the infrared region, is characteristic of polarons and bipolarons in oxidized (doped) PEDOT. When the EDOT ratio was low (*m*(EDOT)/*m*(s-CNF) = 0.2), the absorption tail extending into the infrared region was relatively small, indicating a lower degree of polymerization. These findings, supported by FT-IR and UV-Vis absorption spectra, confirm the successful synthesis of PEDOT:s-CNF.

**Figure 3:**
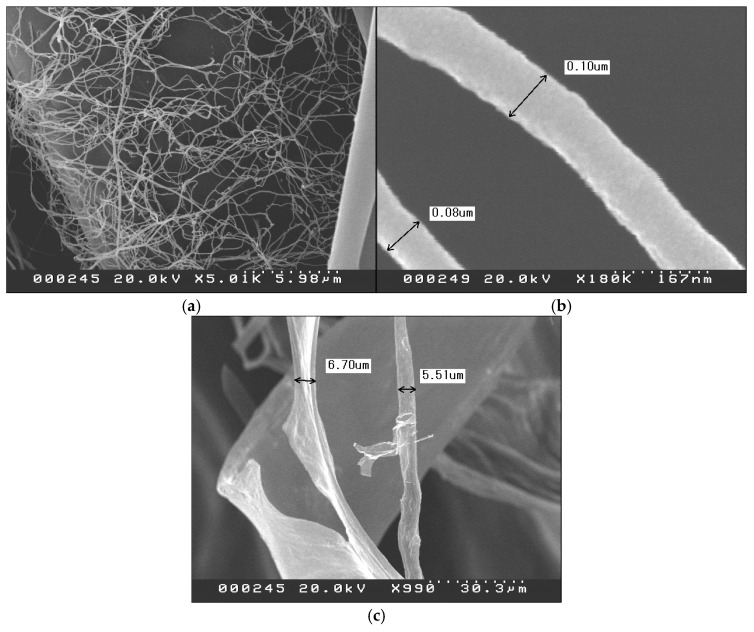
SEM images of (**a**) s-CNF (×5000), (**b**) s-CNF (×180,000), and (**c**) PEDOT:s-CNF (DS of s-CNF = 0.277) with *m*(EDOT)/*m*(s-CNF) = 5.0 (×1000).

Figure 3 presents the SEM images of s-CNF and PEDOT:s-CNF (DS of s-CNF = 0.277, *m*(EDOT)/*m*(s-CNF) = 5.0). The pristine s-CNF exhibited fine nanofiber structures with an approximate thickness of 100 nm. In contrast, PEDOT:s-CNF, where PEDOT polymerization progressed, displayed a significantly increased fiber thickness of approximately 5 µm. This increase in thickness is attributed to the growth of individual nanofibers and the aggregation and association of nanofibers due to polymerization. In PEDOT:PSS, the polymerized PEDOT is covered by hydrophilic and flexible PSS and stabilized in aqueous media, while in PEDOT:s-CNF, the s-CNF molecules form a nanofiber and cannot cover the PEDOT. PEDOT molecules are positioned as if covering the outside of the s-CNF fibers or are percolated into the fibers. As polymerization proceeds, the hydrophilicity of the s-CNF fibers decreases, and the aggregation progresses.

**Figure 4:**
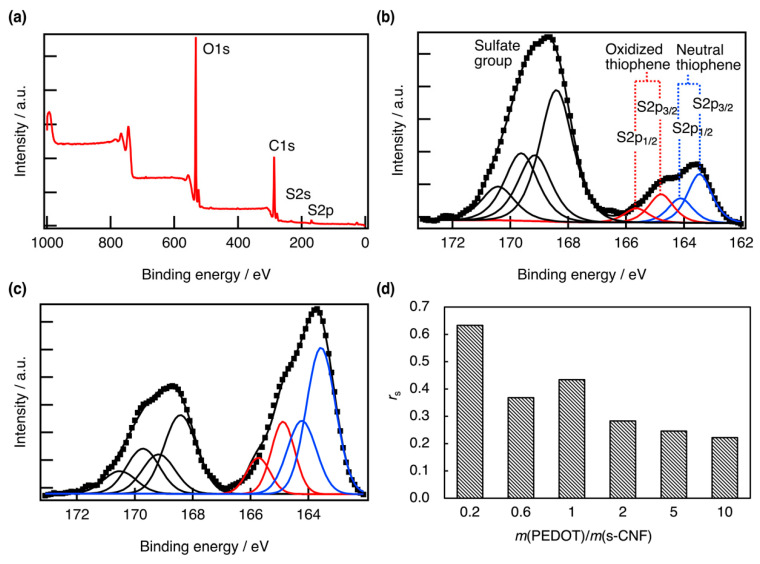
(**a**) XPS spectrum in the wide-range scan of PEDOT:s-CNF (DS of s-CNF = 0.277) with *m*(EDOT)/*m*(s-CNF) of 0.6. The S 2p core-level of PEDOT:s-CNF (DS of s-CNF = 0.277) with *m*(EDOT)/*m*(s-CNF) of (**b**) 0.6 and (**c**) 2.0. (**d**) The area ratio of the doped thiophene peak to the total thiophene peak *r*_s_ with changing *m*(EDOT)/*m*(s-CNF).

Figure 4 presents the XPS core-level spectra of PEDOT:s-CNF (DS of s-CNF = 0.277). Figure 4a shows a wide-range XPS spectrum, confirming the presence of C, O, and S elements for the sample with *m*(EDOT)/*m*(s-CNF) = 0.6. Figure 4b,c display the S 2p core-level spectra of PEDOT:s-CNF at *m*(EDOT)/*m*(s-CNF) = 0.6 and 2.0, respectively. The S 2p core-level spectra for other *m*(EDOT)/*m*(s-CNF) ratios are provided in the Supporting Information (Figure S1). For all samples, the peak in the range of 168–171 eV corresponded to the S 2p of sulfate groups on cellulose, while the peak in the range of 162–166 eV corresponded to the S 2p of the thiophene ring in PEDOT. As *m*(EDOT)/*m*(s-CNF) increased, the intensity of the peak corresponding to S 2p of the thiophene ring increased significantly. Furthermore, the S 2p peak of the thiophene ring consisted of two distinct doublets: oxidized thiophene (S 2p_1_/_2_ and S 2p_3_/_2_), observed in the higher-energy region and neutral thiophene, observed in the lower-energy region [18]. In particular, the former doublet was directly related to the doping concentration, so the area ratio of doped thiophene to total thiophene, denoted as *r*_S_, was defined as follows:

(1) $$r_{S}=\frac{A_{1/2,ox}+A_{3/2,ox}}{A_{1/2,nu}+A_{3/2,nu}+A_{1/2,ox}+A_{3/2,ox}},$$

where $A_{1/2,nu}$, $A_{3/2,nu}$, $A_{1/2,ox}$, and $A_{3/2,ox}$ represent the peak areas of neutral (S_1/2_, S_3/2_) and oxidized (S_1/2_, S_3/2_) thiophene, respectively. Figure 4d shows the variation in *r*_S_ with *m*(EDOT)/*m*(s-CNF). The results indicate that as the mass of PEDOT increased, the ratio of oxidized thiophene to total thiophene decreased. This suggests that the doping concentration decreases with the increasing EDOT content, as the available sulfate groups in s-CNF become insufficient to maintain the doping level. Compared to PSS, which has one sulfonic group for every two carbon atoms in the main chain, cellulose has one sulfate group for every D-glucopyranose unit in the main chain, even at DS = 1, resulting in a concentration that is roughly 2/5 times that of PSS, and these results clarify the problem of an inherently low-doping density.

**Figure 5:**
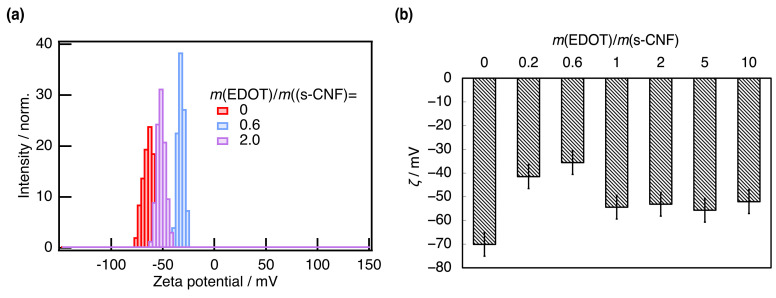
(**a**) The zeta potential distribution of pristine s-CNF (DS of s-CNF = 0.277) and PEDOT:s-CNF with *m*(EDOT)/*m*(s-CNF) = 0.6 and 2.0. (**b**) The zeta potential *ζ* of PEDOT:s-CNF with changing *m*(EDOT)/*m*(s-CNF). The point where *m*(EDOT)/*m*(s-CNF) = 0 means pristine s-CNF.

The doping concentration directly influences the electrical property of PEDOT:s-CNF. As one of the physical values affected by the doping rate of PEDOT:s-CNF, the zeta potential (*ζ*) of the PEDOT:s-CNF (DS of s-CNF = 0.277) aqueous dispersions with changing *m*(EDOT)/*m*(s-CNF) was measured. Figure 5a exhibited the zeta potential distribution of pristine s-CNF and PEDOT:s-CNF and Figure 5b exhibited the *ζ* of PEDOT:s-CNF with changing *m*(EDOT)/*m*(s-CNF). The zeta potential distributions of all measured conditions are presented in Figure S2. The pristine s-CNF exhibited a relatively large absolute zeta potential |*ζ*| of approximately 70 mV. As *m*(EDOT)/*m*(s-CNF) increased, |*ζ*| gradually decreased to approximately 40 mV, indicating that the positive charges on polymerized PEDOT shielded the negative potential of dissociated sulfate groups. However, at higher *m*(EDOT)/*m*(s-CNF) values, |*ζ*| slightly increased again to 50 mV. This trend suggests that the shielding effect weakened as the amount of polymerized PEDOT increased.

**Figure 6:**
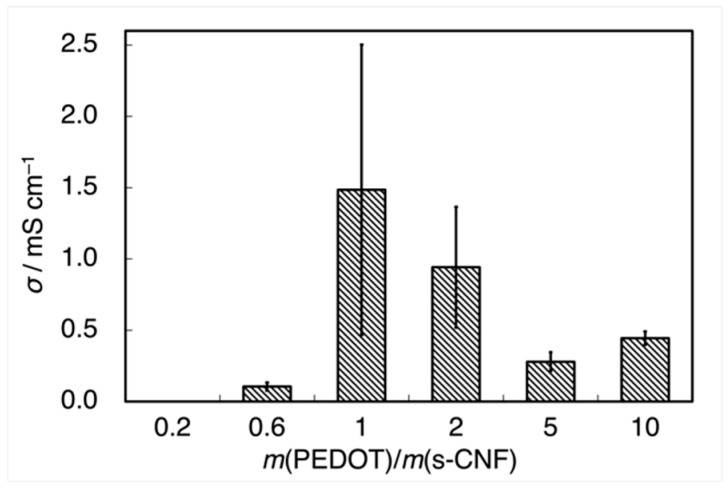
Electrical conductivity of PEDOT:s-CNF (DS of s-CNF = 0.277) with changing *m*(EDOT)/*m*(s-CNF).

Figure 6 illustrates the electrical conductivity (*σ*) of PEDOT:s-CNF (DS = 0.277), measured using the two-probe method as a function of *m*(EDOT)/*m*(s-CNF). Pristine s-CNF and PEDOT:s-CNF (*m*(EDOT)/*m*(s-CNF) = 0.2) were almost insulating, as their current–voltage characteristics could not be reliably measured. As *m*(EDOT)/*m*(s-CNF) increased, *σ* increased to ~1.5 mS cm⁻^1^ at *m*(EDOT)/*m*(s-CNF) = 1.0. However, further increasing *m*(EDOT)/*m*(s-CNF) resulted in a decrease in *σ*, suggesting that the sulfate groups of s-CNF were insufficient to fully dope the EDOT monomers.

Based on the above results, in the low *m*(EDOT)/*m*(s-CNF) region (<1.0), the dissociated sulfate groups on s-CNF were adequately preserved, effectively doping PEDOT. This led to enhanced conductivity while the zeta potential was progressively shielded. However, at higher *m*(EDOT)/*m*(s-CNF) ratios (>1.0), the dissociation of sulfate groups became insufficient, limiting the carrier concentration of PEDOT. Consequently, PEDOT polymerized with a lower carrier density caused volumetric expansion and decreased conductivity.

**Figure 7:**
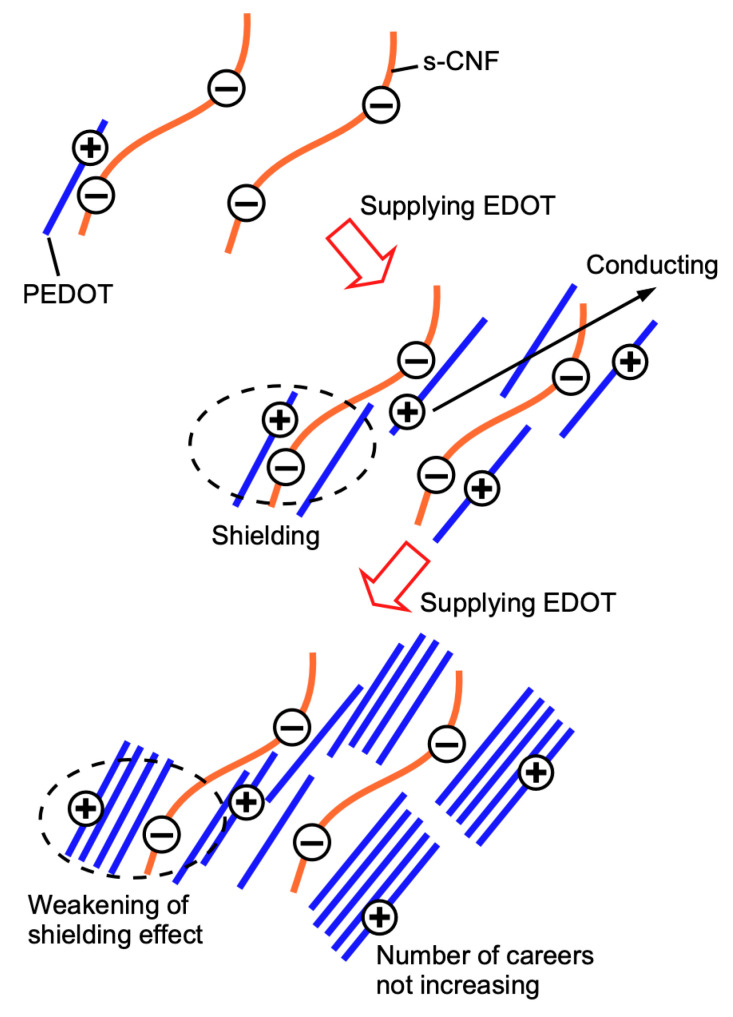
Schematic image of the variation in the conductivity and the zeta potential with increasing *m*(EDOT)/*m*(s-CNF).

Additionally, the reduction in zeta potential shielding at high *m*(EDOT)/*m*(s-CNF) ratios cannot be solely attributed to sulfate depletion. Instead, positive carriers in PEDOT, initially localized around the negative charge of the sulfate groups of s-CNF, likely migrated in aggregated or crystallized PEDOT molecules, thereby relaxing the shielding effect. A schematic representation of the variation in conductivity and zeta potential with increasing *m*(EDOT)/*m*(s-CNF) is shown in Figure 7.

### 3.2. Properties of PEDOT:s-CNF:PTSA

To enhance the conductivity of PEDOT:s-CNF, *p*-toluenesulfonic acid (PTSA) was introduced before the polymerization of EDOT. Since PTSA is known to function as a dopant for PEDOT [19], doping with PTSA can occur independently of the sulfate groups on s-CNF. As a measure of PTSA incorporation, the molar ratio of -SO_3_H (-SO_3_⁻) groups (from both s-CNF and PTSA) to -OH groups on s-CNF was defined as *n*(-SO_3_H)/*n*(-OH). Notably, for PEDOT:s-CNF without PTSA, *n*(-SO_3_H)/*n*(-OH) corresponds to DS = 0.277.

**Figure 8:**
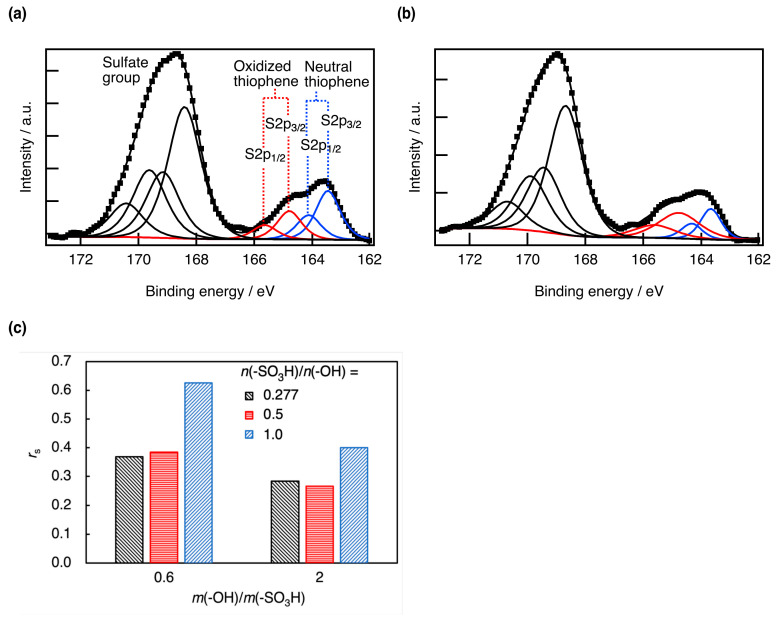
The S 2p core-level of PEDOT:s-CNF:PTSA (DS of s-CNF = 0.277 and *m*(EDOT)/*m*(s-CNF) = 0.6) with *n*(-SO_3_H)/*n*(-OH) at (**a**) 0.277 and (**b**) 1.0. (**c**) The area ratio of the doped thiophene peak to the total thiophene peak *r*_s_ of PEDOT:s-CNF:PTSA (DS of s-CNF = 0.277) and *m*(EDOT)/*m*(s-CNF) = 0.6 and 2.0, estimated by the XPS core-level of S 2p with changing *n*(-SO_3_H)/*n*(-OH).

The XPS core-level spectrum of S 2p in PEDOT:s-CNF:PTSA (DS = 0.277, *m*(EDOT)/*m*(s-CNF) = 0.6) is shown in Figure 8a,b, and the area ratio *r*_S_ of doped to total thiophene is presented in Figure 8c. In addition, the core-level spectrum of S 2p in PEDOT:s-CNF:PTSA with other *m*(EDOT)/*m*(s-CNFs) is shown in Figure S3. With the addition of PTSA (increasing *n*(-SO_3_H)/*n*(-OH)), *r*_S_ exhibited an increasing trend, regardless of *m*(EDOT)/*m*(s-CNF). This suggests that the sulfonic groups in PTSA effectively functioned as dopants. PEDOT doped with PTSA has been reported to exhibit higher conductivity than that doped with PSS [19], and it remains a superior dopant in PEDOT:s-CNF. In addition, the peaks corresponding to the oxidized thiophene are broadened by the addition of PTSA regardless of *m*(EDOT)/*m*(s-CNF). This broadening is suggested to indicate some interaction between PEDOT and PTSA.

**Figure 9:**
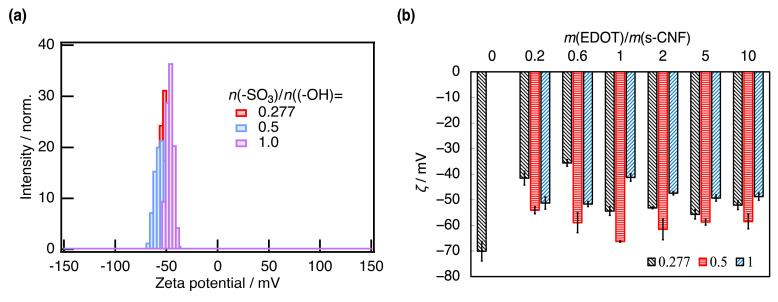
(**a**) The zeta potential distribution of PEDOT:s-CNF:PTSA (DS of s-CNF = 0.277 and *m*(EDOT)/*m*(s-CNF) = 2.0) with *n*(-SO_3_H)/*n*(-OH) = 0.277, 0.5, and 1.0. (**b**) The zeta potential *ζ* of PEDOT:s-CNF:PTSA with changing *m*(EDOT)/*m*(s-CNF) and *n*(-SO_3_H)/*n*(-OH).

Figure 9a exhibits the zeta potential distribution of PEDOT:s-CNF:PTSA (DS of s-CNF = 0.277 and *m*(EDOT)/*m*(s-CNF) = 2.0) and Figure 9b exhibits the *ζ* of PEDOT:s-CNF:PTSA as a function of both *m*(EDOT)/*m*(s-CNF) and *n*(-SO_3_H)/*n*(-OH). The zeta potential distributions of all measured conditions are presented in Figures S4–S6. When PTSA was first added (*n*(-SO_3_H)/*n*(-OH) = 0.5), |*ζ*| increased. This increase was attributed to PTSA molecules doping PEDOT and attaching to the PEDOT:s-CNF surface, thereby increasing |*ζ*|. Furthermore, excess PTSA molecules, even if not actively contributing to doping, may still adhere to the surface of PEDOT:s-CNF, further elevating |*ζ*|. However, beyond a certain threshold, additional PTSA led to a decrease in |*ζ*|. This reduction is likely due to the PTSA molecules already attaching to the surface of PEDOT:s-CNF, preventing additional PTSA molecules from approaching the PEDOT:s-CNF. Additionally, excess PTSA lowers pH, reducing the degree of sulfate group dissociation on s-CNF or hydrolyzing the sulfate group in extreme cases, thereby diminishing doping efficiency and decreasing |*ζ*|.

**Figure 10:**
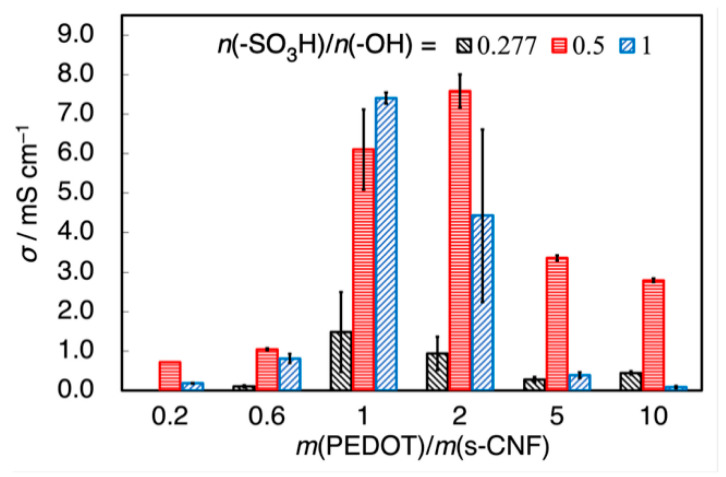
Conductivity *σ* of PEDOT:s-CNF:PTSA (DS of s-CNF = 0.277) with changing *m*(EDOT)/*m*(s-CNF) and *n*(-SO_3_H)/*n*(-OH).

Figure 10 illustrates the conductivity (*σ*) of PEDOT:s-CNF:PTSA as a function of *m*(EDOT)/*m*(s-CNF) and *n*(-SO_3_H)/*n*(-OH). The introduction of PTSA resulted in a significant improvement in conductivity. When *m*(EDOT)/*m*(s-CNF) was 1.0 or 2.0, the conductivity of PEDOT:s-CNF:PTSA reached values close to 10 mS cm⁻^1^, demonstrating the effectiveness of PTSA doping. However, the conductivity declined when excessive PTSA was added, particularly in the high *m*(EDOT)/*m*(s-CNF) region (greater than 5.0). The primary reason for this decrease is that sulfate groups on s-CNF became insufficient for EDOT doping in this region, reducing the potential conductivity enhancement from PTSA. Moreover, because sulfate groups are weaker acids than sulfonic groups, the decrease in pH due to excessive PTSA further suppressed sulfate group dissociation, lowering the doping efficiency of s-CNF and contributing to the decline in *σ.*

Another factor contributing to this decrease in conductivity is the intrinsic brittleness of PEDOT:s-CNF films. Excessive PTSA, along with a significant drop in pH, may lead to structural instability, making the film more prone to critical defects that negatively impact electrical performance.

Since *n*(-SO_3_H)/*n*(-OH) = 1.0 corresponds to PEDOT:s-CNF with a DS value of 1.0, increasing the DS value of s-CNF appears to be a viable strategy for further enhancing conductivity, even though the acid dissociation constants of sulfate and sulfonic groups differ.

### 3.3. PEDOT:s-CNF Dispersion Using s-CNF with Different DSs

The increase in conductivity observed with the addition of PTSA suggests that the sulfate groups present on s-CNFs were insufficient and that PEDOT remained receptive to further doping. This indicates that increasing the concentration of sulfate groups on s-CNF should enhance the conductivity of PEDOT. To explore this effect, s-CNF with a higher degree of substitution (DS = 0.343) was investigated.

The XPS core-level spectrum of S 2p for PEDOT:s-CNF at *m*(EDOT)/*m*(s-CNF) = 2.0 was analyzed to determine the area ratio *r*_S_ of doped to total thiophene. When using s-CNF with DS = 0.343, *r*_S_ was estimated to be 0.300, which was slightly higher than the value observed for DS = 0.277 (*r*_S_ = 0.283, Figure S7). While the difference was minor, it suggests that a higher DS value promotes doping in PEDOT.

**Figure 11:**
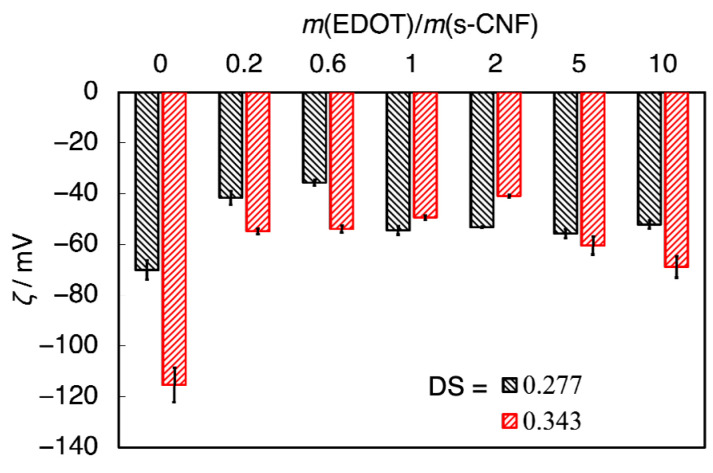
The zeta potential *ζ* of PEDOT:s-CNF prepared using s-CNF with different DSs.

The impact of DSs on zeta potential was examined (Figure S8). Figure 11 illustrates the zeta potential of PEDOT:s-CNF prepared using s-CNF with different DS values. In the low *m*(EDOT)/*m*(s-CNF) region (<0.6), s-CNF with a higher DS exhibited a larger absolute zeta potential (|*ζ*|). This behavior is attributed to the presence of excess sulfate groups, which generated greater negative potential in conditions where the number of sulfate groups exceeded that of EDOT molecules. However, in the high *m*(EDOT)/*m*(s-CNF) region (>1.0), where an excess of EDOT molecules was available, sulfate groups on s-CNF became shielded by positively doped PEDOT. As a result, the zeta potential for different DS values converged at approximately ~50 mV, with no significant differences between the samples.

**Figure 12:**
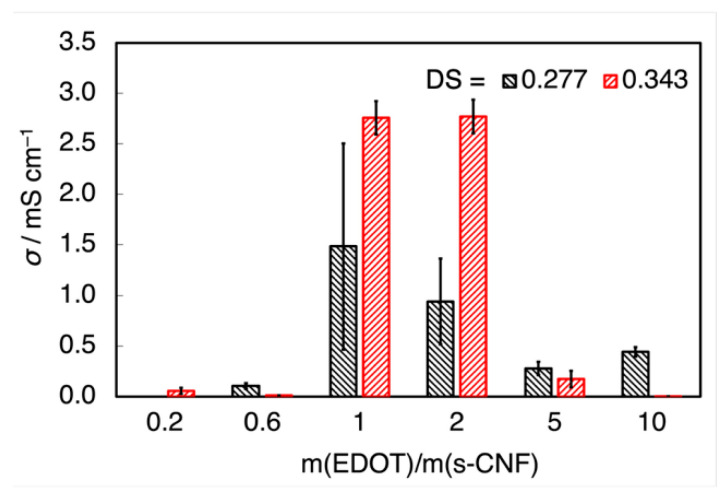
The conductivity of the PEDOT: s-CNF film prepared using s-CNF with different DSs.

The relationship between DS and conductivity was further investigated. Figure 12 illustrates the conductivity (*σ*) of PEDOT:s-CNF prepared using s-CNF with different DS values. Under the conditions of *m*(EDOT)/*m*(s-CNF) = 1.0 or 2.0, where conductivity was relatively high, the s-CNF sample with a larger DS exhibited increased conductivity. The highest conductivity, approximately 3.0 mS cm⁻^1^, was achieved with DS = 0.343. These results indicate that s-CNF with a high DS value provides an advantage in achieving high electrical conductivity. This enhancement can be attributed to the increased concentration of sulfate groups, which improved the doping efficiency of PEDOT. The trend is consistent with prior observations, further reinforcing the correlation between sulfate group density and doping effectiveness.

Considering the overall results, the polymerization of PEDOT was initiated by adding EDOT, leading to an increase in conductivity when the number of EDOT molecules remained lower than the number of available sulfate groups on s-CNF. However, because the sulfate group density on s-CNF was substantially lower than that on PSS, achieving adequate doping became increasingly difficult as the EDOT concentration increased. Consequently, the excessive addition of EDOT resulted in a decline in conductivity. To counteract this effect, strategies such as increasing the degree of substitution or incorporating additional compounds containing sulfonic groups to enhance the dopant concentration have been proposed. The addition of PTSA proved to be an effective means of improving conductivity. However, at higher PTSA concentrations, the strong acidity of the sulfonic groups reduced the degree of sulfate group dissociation, leading to a decline in doping efficiency.

## 4. Conclusions

PEDOT polymerized on sulfated cellulose nanofiber (PEDOT:s-CNF) was successfully synthesized, and the relationship between conductivity and the doping state of PEDOT:s-CNF was investigated. The optimal amount of the EDOT monomer required to maximize conductivity (~1.5 mS cm^−1^) is determined by the number of sulfate groups of s-CNF. The excessive polymerization reduced electrical conductivity by the depletion of sulfate groups, which acted as dopants on s-CNFs. On the other hand, the reduction in zeta potential shielding during excessive polymerization could not be solely attributed to the depletion of sulfate groups. The positive carriers in PEDOT initially localized around the negative charge of the sulfate groups of s-CNF and migrated in aggregated or crystallized PEDOT molecules, thereby relaxing the shielding effect. While the addition of other dopants containing sulfonic groups was an effective approach to compensate for low DS values and enhance conductivity (~10 mS cm^−1^), their excessive addition resulted in conductivity suppression due to differences in the acidity of sulfate and sulfonic groups. To achieve high conductivity, increasing the density of sulfate groups is necessary, so s-CNFs with high DS values also had an advantage. The results indicate the possibility of replacing the PSS of PEDOT:PSS with bio-based materials.

## Data Availability

The original contributions presented in this study are included in the article and Supplementary Material. Further inquiries can be directed to the corresponding author.

## Supplementary Materials

The following supporting information can be downloaded at https://www.mdpi.com/article/10.3390/ma18061273/s1. Figure S1: X-ray photoelectron spectroscopy of PEDOT:s-CNF (DS of s-CNF = 0.277); Figure S2: Electrophoretic light scattering of PEDOT:s-CNF (DS of s-CNF = 0.277); Figure S3: X-ray photoelectron spectroscopy of PEDOT:s-CNF:PTSA (DS of s-CNF = 0.277); Figures S4–S6: Electrophoretic light scattering of PEDOT:s-CNF:PTSA (DS of s-CNF = 0.277); Figure S7: X-ray photoelectron spectroscopy of PEDOT:s-CNF with different DSs; Figure S8: Electrophoretic light scattering of PEDOT:s-CNF (DS of s-CNF = 0.343).

## Abbreviations

The following abbreviations are used in this manuscript:

PEDOT	Poly(3,4-ethylenedioxythiophene)
PSS	Poly(styrene sulfonate)
CNF	Cellulose nanofiber
s-CNF	Sulfated cellulose nanofiber
PTSA	*p*-Toluenesulfonic acid
DBSA	Dodecylbenzenesulfonic acid
EDOT	3,4-Ethylenedioxythiophene
DS	Degree of substitution
SNC	Nanocrystalline cellulose
KPS	Potassium persulfate
FT-IR	Fourier transform-infrared spectroscopy
UV-Vis	Ultraviolet–visible absorption
SEM	Scanning electron microscopy
XPS	X-ray photoelectron spectroscopy
