# Supplementary material for: Polymerization of Poly(3,4-ethylenedioxythiophene) on Sulfated Cellulose Nanofiber and Its Conducting Property
Source: Materials (Basel). 2025 Mar 13;18(6):1273. doi: 10.3390/ma18061273 (PMC11943885; doi:10.3390/ma18061273)
Supplement: Supplementary file 1 [file materials-18-01273-s001.zip › materials-3514684-supplementary.pdf]

materials

MDPI

Supplementary Materials

Polymerization of Poly(3,4-Ethylenedioxythiophene) on Sulfated Cellulose Nanofiber and Its Conducting Property

Naofumi Takahashi, Atsuya Ogo and Takeshi Shimomura *

Graduate School, Tokyo University of Agriculture and Technology, 2-24-16 Naka-cho, Koganei, Tokyo 184-8588, Japan
* Correspondence: simo@cc.tuat.ac.jp; Tel.: +81-42-388-7051

A. X-ray photoelectron spectroscopy of PEDOT:s-CNF (DS of s-CNF = 0.277)

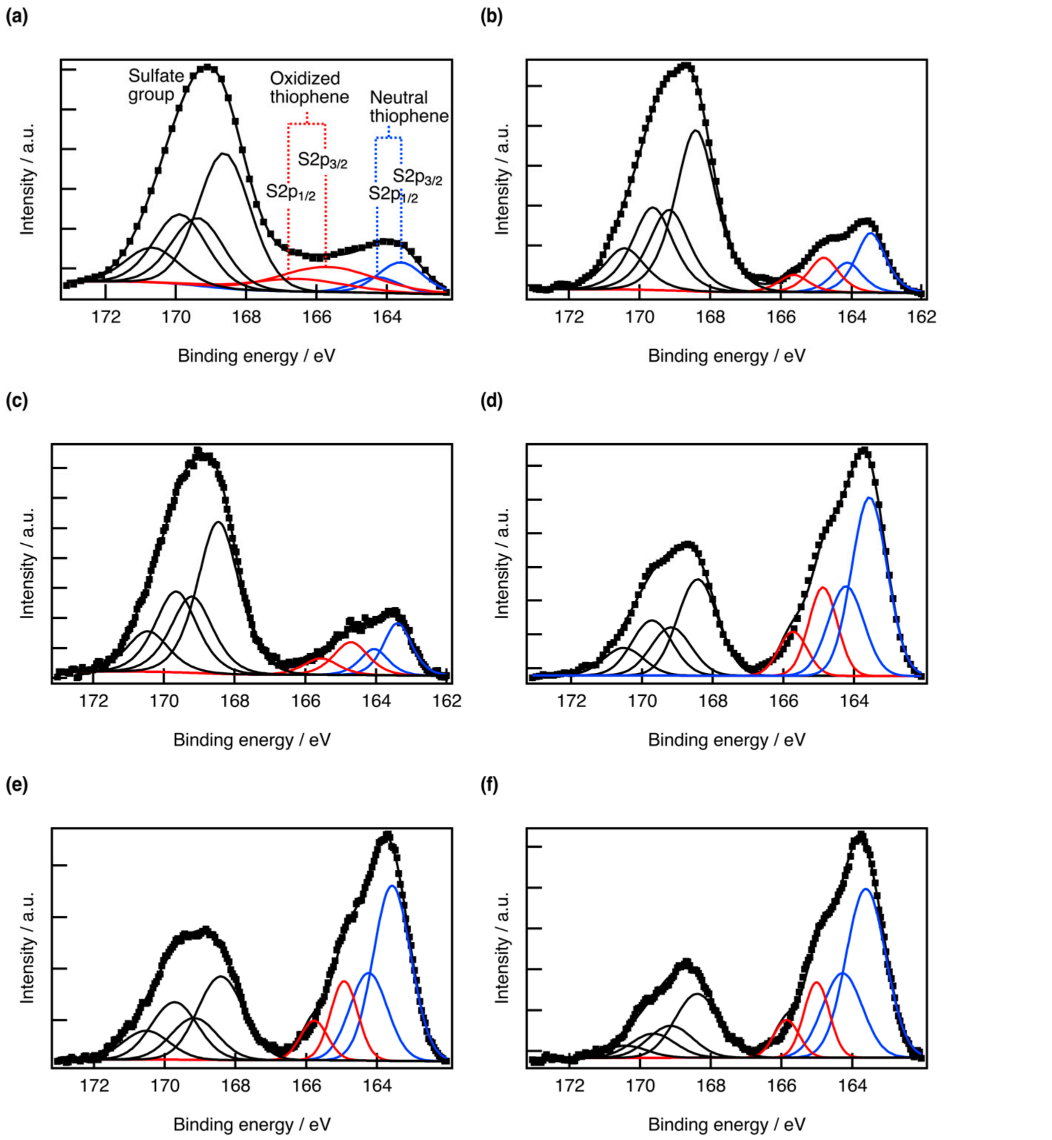

Figure S1. The XPS S 2p core-level of PEDOT:s-CNF (DS of s-CNF = 0.277) with *m*(EDOT)/*m*(s-CNF) = (a) 0.2, (b) 0.6, (c) 1.0, (d) 2.0 and (e) 10.

B. Electrophoretic light scattering of PEDOT:s-CNF (DS of s-CNF = 0.277)

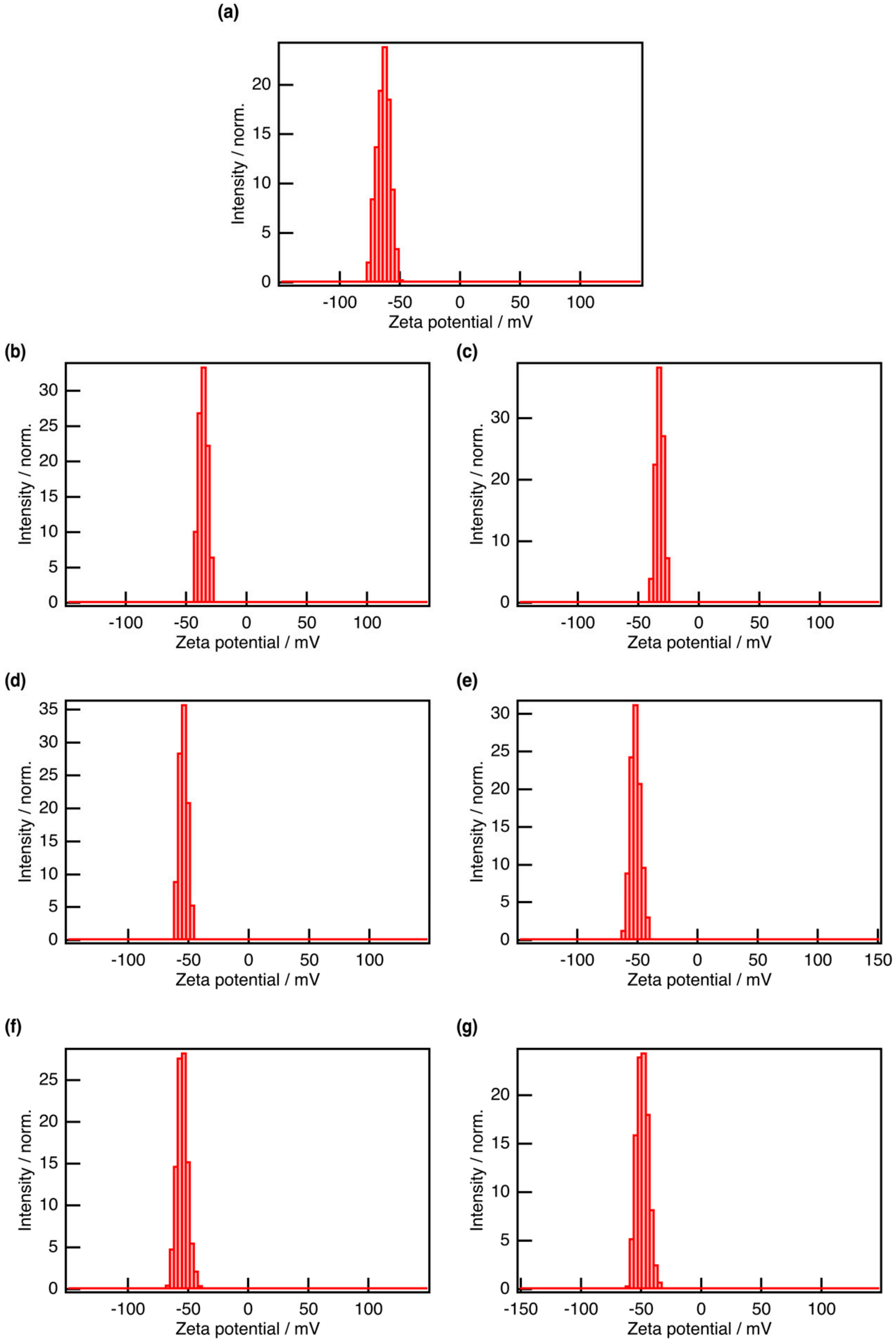

Figure S2. The zeta potential distribution of (a) pristine s-CNF (DS of s-CNF = 0.277) and PEDOT:s-CNF with *m*(EDOT)/*m*(s-CNF) = (b) 0.2, (c) 0.6, (d) 1.0, (e) 2.0 and (f) 10.

C. X-ray photoelectron spectroscopy of PEDOT:s-CNF:PTSA (DS of s-CNF = 0.277)

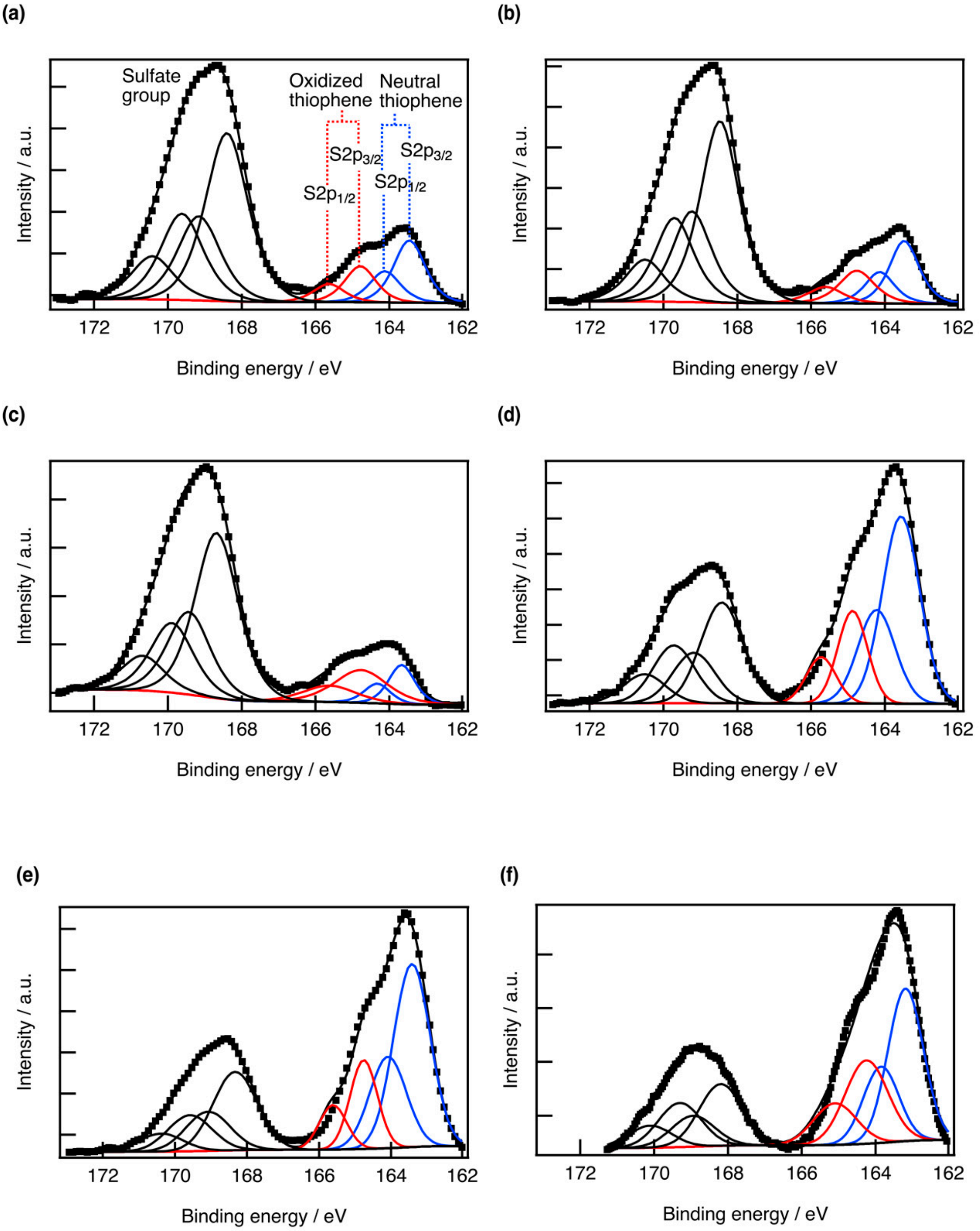

Figure S3. The XPS S 2p core-level of PEDOT:s-CNF:PTSA (DS of s-CNF = 0.277) with changing *n*(-SO_3H)/*n*(-OH). (a) The PEDOT:s-CNF and PEDOT:s-CNF:PTSA with *n*(-SO_3H)/*n*(-OH) to be (b) 0.5, (c) 1.0 with *m*(EDOT)/*m*(s-CNF) = 0.6, and (d) the PEDOT:s-CNF and PEDOT:s-CNF:PTSA with *n*(-SO_3H)/*n*(-OH) to be (e) 0.5, (f) 1.0 with *m*(EDOT)/*m*(s-CNF) = 2.0.

D. Electrophoretic light scattering of PEDOT:s-CNF:PTSA (DS of s-CNF = 0.277)

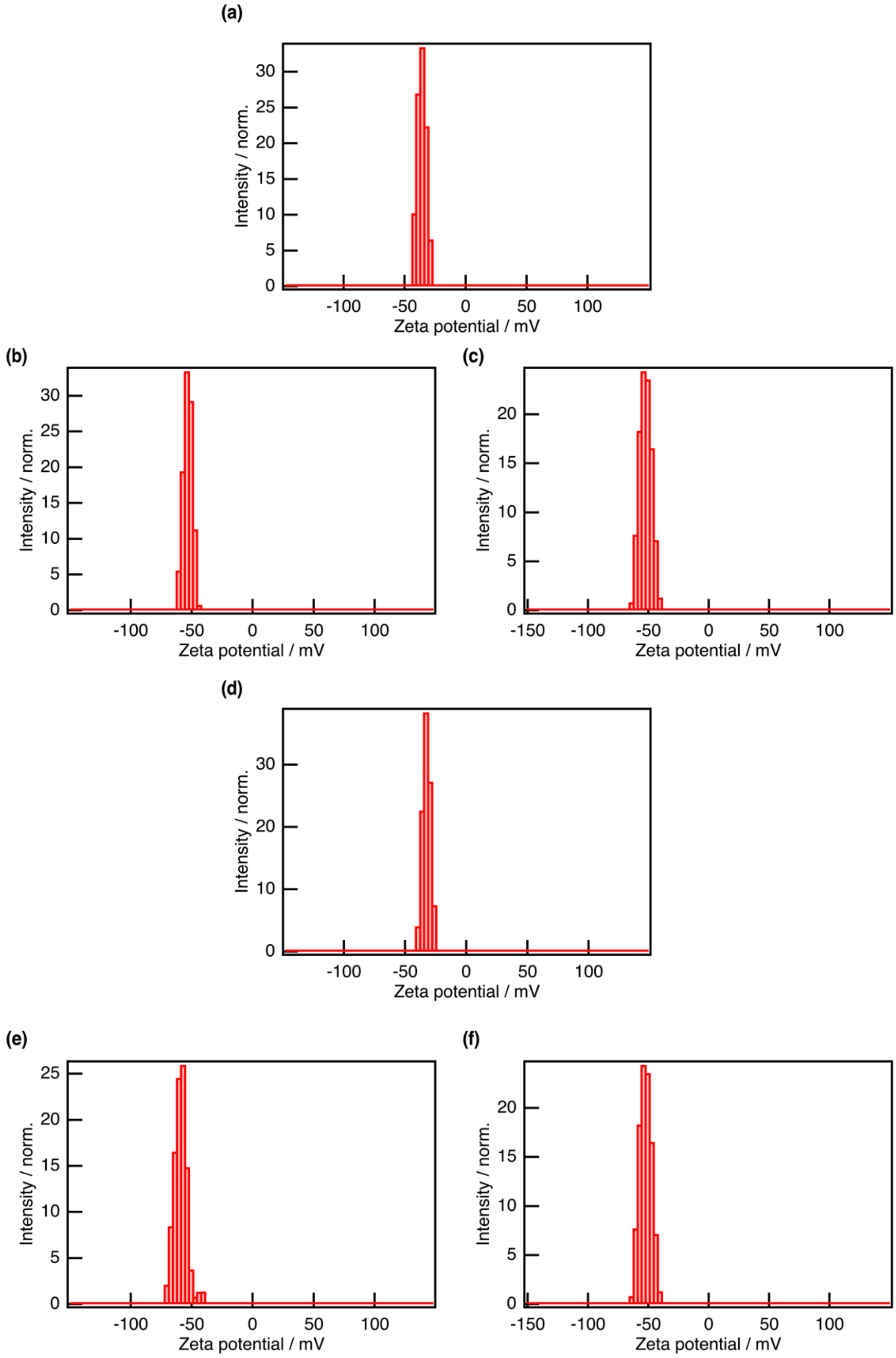

Figure S4. The zeta potential distribution of PEDOT:s-CNF:PTSA (DS of s-CNF = 0.277) with changing *n*(-SO_3H)/*n*(-OH). (a) The PEDOT:s-CNF and PEDOT:s-CNF:PTSA with *n*(-SO_3H)/*n*(-OH) to be (b) 0.5, (c) 1.0 with *m*(EDOT)/*m*(s-CNF) = 0.2, and (d) the PEDOT:s-CNF and PEDOT:s-CNF:PTSA with *n*(-SO_3H)/*n*(-OH) to be (e) 0.5, (f) 1.0 with *m*(EDOT)/*m*(s-CNF) = 0.6.

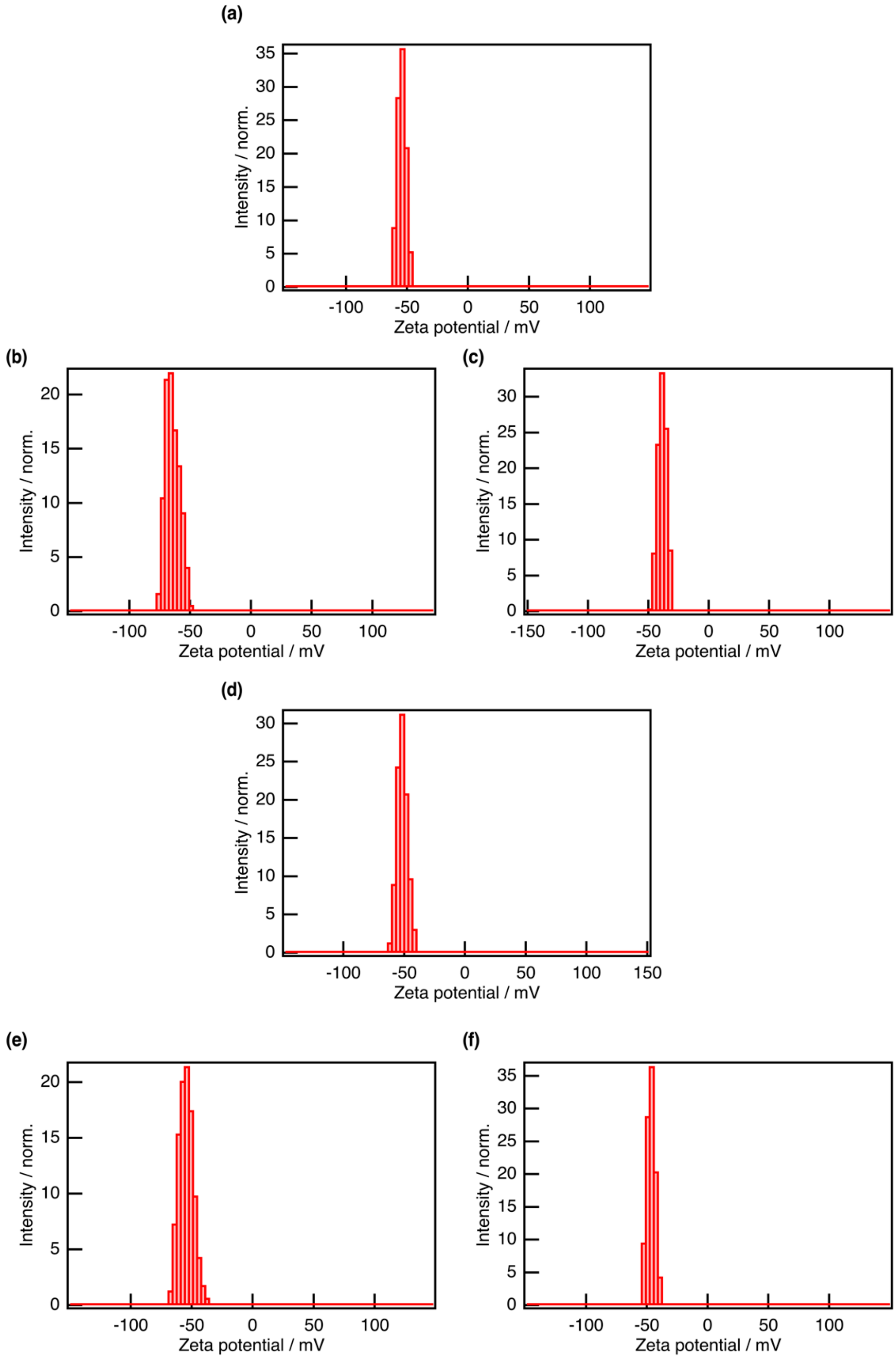

Figure S5. The zeta potential distribution of PEDOT:s-CNF:PTSA (DS of s-CNF = 0.277) with changing n(-SO_3H)/n(-OH). (a) The PEDOT:s-CNF and PEDOT:s-CNF:PTSA with n(-SO_3H)/n(-OH) to be (b) 0.5, (c) 1.0 with m(EDOT)/m(s-CNF) = 1.0, and (d) the PEDOT:s-CNF and PEDOT:s-CNF:PTSA with n(-SO_3H)/n(-OH) to be (e) 0.5, (f) 1.0 with m(EDOT)/m(s-CNF) = 2.0.

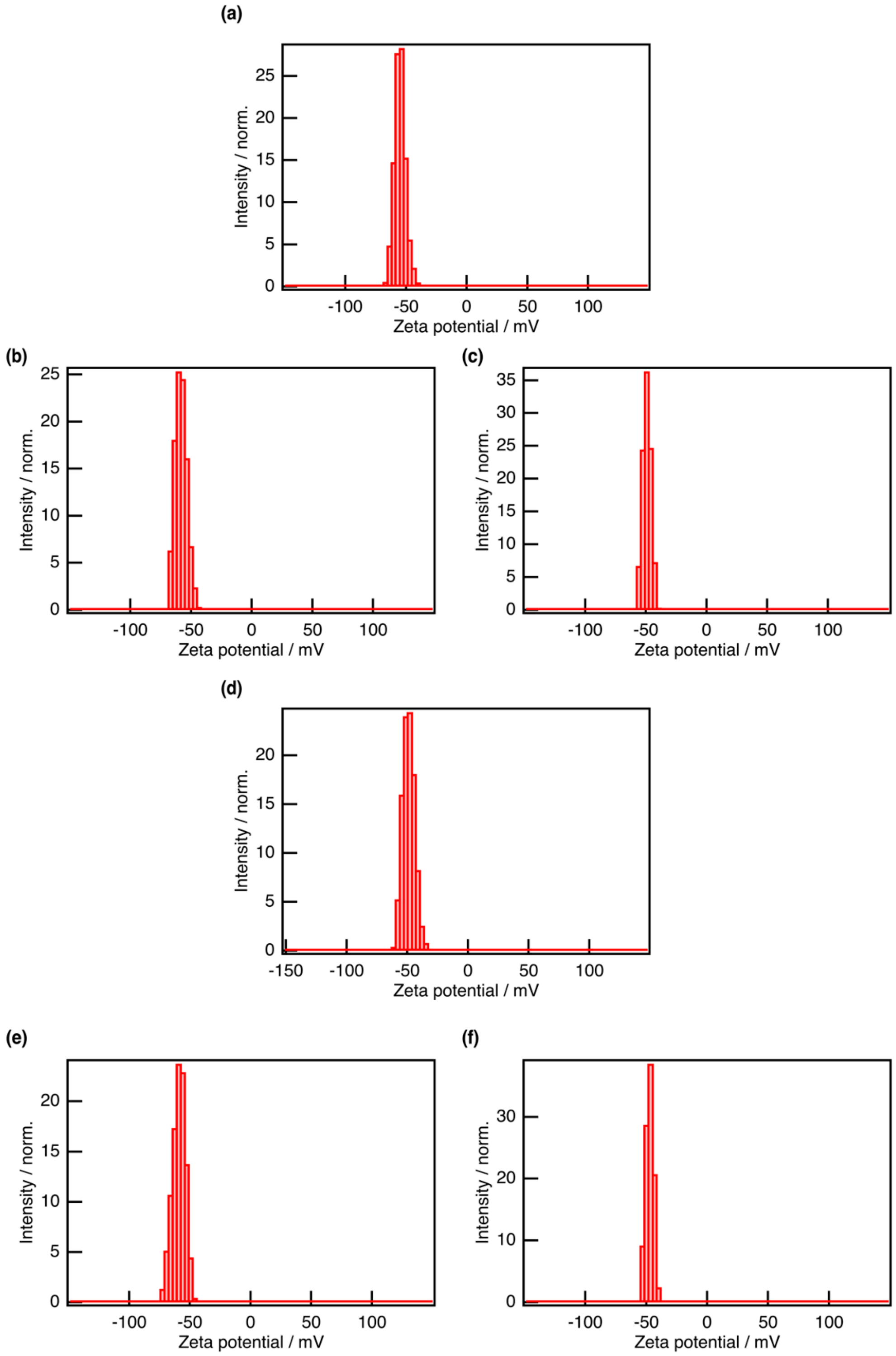

Figure S6. The zeta potential distribution of PEDOT:s-CNF:PTSA (DS of s-CNF = 0.277) with changing n(-SO_3H)/n(-OH). (a) The PEDOT:s-CNF and PEDOT:s-CNF:PTSA with n(-SO_3H)/n(-OH) to be (b) 0.5, (c) 1.0 with m(EDOT)/m(s-CNF) = 5.0, and (d) the PEDOT:s-CNF and PEDOT:s-CNF:PTSA with n(-SO_3H)/n(-OH) to be (e) 0.5, (f) 1.0 with m(EDOT)/m(s-CNF) = 10.

E. X-ray photoelectron spectroscopy of PEDOT:s-CNF with different DS

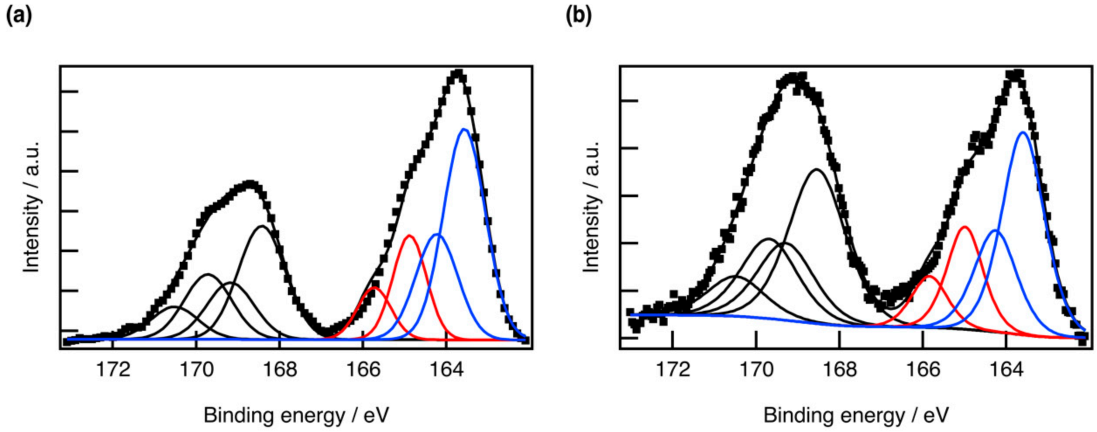

Figure S7. The XPS S 2p core-level of PEDOT:s-CNF at *m*(EDOT)/*m*(s-CNF) = 2.0 with changing DS of s-CNF. (a) DS = 0.277 and (b) 0.343.

F. Electrophoretic light scattering of PEDOT:s-CNF (DS of s-CNF = 0.343)

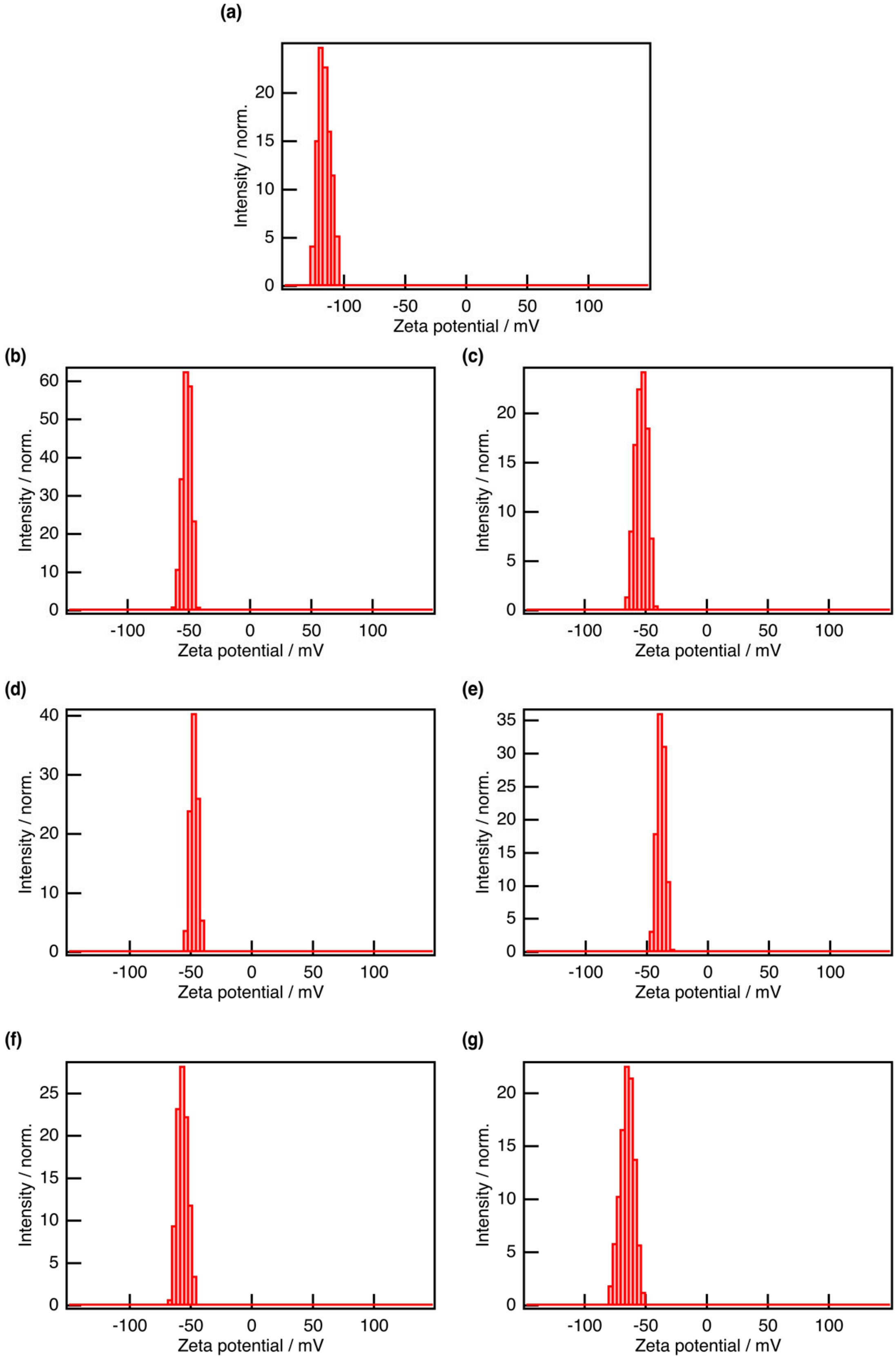

Figure S8. The zeta potential distribution of (a) pristine s-CNF (DS of s-CNF = 0.343) and PEDOT:s-CNF with *m*(EDOT)/*m*(s-CNF) = (b) 0.2, (c) 0.6, (d) 1.0, (e) 2.0 and (f) 10 measured by ELS.
